# The Antidepressant Mirtazapine Activates Hepatic Macrophages, Facilitating Pathogen Clearance While Limiting Tissue Damage in Mice

**DOI:** 10.3389/fimmu.2020.578654

**Published:** 2020-11-03

**Authors:** Rachelle Paige Davis, Wagdi Almishri, Craig Neal Jenne, Mark Gordon Swain

**Affiliations:** ^1^ Department of Microbiology, Immunology, and Infectious Diseases, University of Calgary, Calgary, AB, Canada; ^2^ Snyder Institute for Chronic Diseases, University of Calgary, Calgary, AB, Canada

**Keywords:** mirtazapine, imaging, macrophage, inflammation, infection, liver

## Abstract

**Background and Aims:**

Mirtazapine is an atypical antidepressant with antagonist activity for serotonin and histamine receptors. Clinical and experimental evidence suggests that, in addition to treating depression, mirtazapine also alters liver innate immunity and suppresses immune-driven hepatic macrophage activation. Liver macrophages, Kupffer cells, represent the largest collection of fixed macrophages in the body and are critical in regulating hepatic immunity. In addition to their capacity to regulate inflammation, Kupffer cells are key sentinels for clearing blood-borne pathogens, preventing their dissemination within the body. This process involves pathogen capture, phagocytosis, and activation-induced killing *via* reactive oxygen species (ROS) production. Therefore, we speculated that mirtazapine might adversely alter Kupffer cell pathogen-associated activation and killing.

**Methods:**

Mice were treated with mirtazapine and time-dependent changes in Kupffer cells were characterized using intravital microscopy. Macrophage and neutrophil responses, bacterial dissemination, and liver damage were assessed following i.v. infection with a pathogenic strain of *S. aureus*.

**Results:**

Mirtazapine rapidly (within 1.5 h) activates Kupffer cells, indicated by a loss of elongated shape with cellular rounding. However, this shape change did not result in impaired pathogen capture function, and, in fact, generated enhanced ROS production in response to *S. aureus*-induced sepsis. Neutrophil dynamics were altered with reduced cellular recruitment to the liver following infection. Bacterial dissemination post-intravenous administration was not altered by mirtazapine treatment; however, hepatic abscess formation was significantly reduced.

**Conclusions:**

Mirtazapine rapidly activates Kupffer cells, associated with preserved bacterial capture functions and enhanced ROS generation capacity. Moreover, these changes in Kupffer cells were linked to a beneficial reduction in hepatic abscess size. In contrast to our initial speculation, mirtazapine may have beneficial effects in sepsis and warrants further exploration.

## Highlights

Treatment of animals with the atypical antidepressant mirtazapine activates liver macrophages. This activation enhances the ability of these cells to kill bacteria while simultaneously reducing the overall inflammatory response. This liver reprogramming results in an efficient immune response and pathogen clearance while limiting inflammation-mediate collateral damage to the liver.

## Introduction

Depression is common in society, with a global lifetime prevalence of up to 15% and significant associated disability (1, 2). In addition, the risk of having depression increases significantly in people with one or more co-morbid chronic diseases (3), and co-existing depression has been reported to worsen the clinical course of many of these chronic conditions (4–6). The use of antidepressants is common among patients with comorbid medical conditions (7) and interestingly, antidepressant therapy may improve clinical outcomes in these patients (8–10). However, despite these clinical observations there is limited understanding of the impact of antidepressants on immunity.

Recently, using a large epidemiological database, we identified a beneficial effect of the atypical antidepressant mirtazapine on adverse clinical liver outcomes in patients with the autoimmune liver disease primary biliary cholangitis (10). Mirtazapine, classified as an atypical antidepressant, is commonly prescribed for the treatment of major depression. Mirtazapine exhibits promiscuous receptor engagement, including antagonistic effects at α2-adrenoreceptors and 5-HT_2_ and HT_3_ receptors (11). Interestingly, these receptors are particularly enriched in the liver, especially on macrophages (12). In translational bench research, we used a mouse model of immune-mediated liver injury to define mechanism(s), replicating many clinical observations from patients with autoimmune liver disease, and found that mirtazapine had a marked impact on hepatic innate-immune activation. Specifically, we found that mirtazapine treatment suppressed hepatic macrophage activation and cytokine/chemokine release in this model, in association with decreased hepatic neutrophil recruitment (12).

Although the inflammation-suppressive effect of mirtazapine in experimental immune-driven liver injury was beneficial in attenuating liver damage, hepatic macrophages also have well-defined and critical roles in the capture and killing of pathogenic bacteria within the circulation, including *S. aureus* (13). This biological action of macrophages located within hepatic sinusoids is key for preventing bacterial seeding of the liver and associated liver damage, and in stopping systemic dissemination of bacteria entering the circulation (14, 15). Therefore, we speculated that a broad suppressive effect of mirtazapine on macrophage function could potentially have a detrimental impact on the capture and killing of bacteria within the circulation. To examine this possibility, we used the well-characterized mouse model of *S. aureus*-induced sepsis and intravital microscopy (IVM) (13, 16, 17) to delineate the impact of mirtazapine on hepatic macrophage bacterial capture and killing.

## Materials and Methods

### Mice

C57Bl/6 mice were purchased from Jackson Laboratories (age 8–10 weeks; Bar Harbor, ME, USA). Animals were housed in a pathogen-free environment at the University of Calgary. All experimental protocols were approved by the University of Calgary Animal Care Committee and were in compliance with guidelines from the Canadian Council for Animal Care (AC18-0050).

### Antibodies and Reagents

Antibodies and fluorescent probes are listed in **Supplementary Table 1**. pHrodo™ Red S. aureus Bioparticles™ were counter-labeled using an Alexa Fluor™ 647 Antibody labelling kit (Thermofisher). Liver perfusion was assessed following i.v. injection of FITC-dextran and enumeration of occluded liver sinusoids.

### Intravital Microscopy (IVM)

Surgical preparation of animals for intravital microscopy of the mouse liver was performed as previously described (18). After general anesthesia (10 mg/kg xylazine hydrochloride and 200 mg/kg ketamine hydrochloride), an i.v. catheter was inserted in the tail vein to inject fluorescently labeled antibodies, bacteria, or additional anesthetic directly into the bloodstream. For surgery, a laparotomy was performed, and the abdominal skin and peritoneum were removed to expose the liver. The falciform ligament was cut after securing the sternum away from the liver using a suture. The mouse was moved to a heated stage, to maintain body temperature throughout image acquisition, and placed on its right side. Using a wet cotton swab, the stomach was manipulated to maneuver the liver into place on a glass coverslip. The gastrointestinal tract was moved away from the liver and secured by wrapping in wet gauze. One layer of wet tissue was placed on the liver to preserve physiological conditions, prevent drying, and diminish movement. Body temperature was maintained *via* heated stage throughout image acquisition. Imaging was performed using an inverted Leica SP8 resonance-scanning microscope (Leica Microsystems) with a 25× water-immersion objective lens.

### Image Analysis

Still images from single channel fluorescence (platelets, neutrophil elastase, FITC contrast agent, neutrophils, Kupffer cells) were exported from acquisition software (Leica LasX) as.tiff images. Minimum threshold values were applied to decrease background fluorescence signal. The same threshold values were applied to images from all treatment groups within a single experiment. For Kupffer cells (KCs), images were imported into image J and every F4/80+ cell was measured for the following parameters: roundness (scale = 0–1, where 1 is perfect circularity), percent area (per field of view [FOV]), average cell size (μm^2^), and the number of cells/FOV (3 FOV/animal, 5–7 animals/group).

For platelet aggregates, images were imported into Image J and each FOV was analyzed for number of platelet aggregates greater-than-or-equal-to each indicated size (μm^2^). Number of aggregates were enumerated and averaged for 3 FOV/animal, 4–5 animals/group. Neutrophil elastase area was assessed as percent area of a FOV labeled with anti-NE antibody (3 FOV/animal, 8 animals/group). For microvascular perfusion measurement, vessels containing no fluorescent signal after 2 min were considered a non-perfused vessel; the number of occluded vessels were counted manually/FOV (3 FOV/animal, 3 animals/group). Neutrophils were counted manually as number of Ly6G+ cells/FOV (3 FOV/animal, 4–5 animals/group).

KC and second signal colocalization quantification: Beads/*S. aureus* particles and KC number per FOV were counted manually. The number of F4/80+ cells with a captured bead/particle was divided by the total number of F4/80+ cells per FOV to account for variance of the number of KCs between each FOV. The same methodology was applied to ROS and pHrodo signals to determine the percentage of F4/80+ cells exhibiting these fluorescent signals (3 FOV/animal, 3–10 animals/group).

### Histology

Liver samples were collected and put into formalin for fixation. After embedding in paraffin, 4.0 μm sections were stained with hematoxylin and eosin (H&E). Images were taken with a Leica Aperio AT2 scanscope microscope, and then analyzed using Image J software (ImageJ, U. S. National Institutes of Health, Bethesda, Maryland, USA, https://imagej.nih.gov/ij) to quantify the extent of liver cell necrosis (necrotic area of liver/total area examined and number of lesions per section);10 randomly selected high-power images measured/mouse).

### Bacterial Culture and Infection

For *Staphylococcus aureus* infections, bacteria (USA300) was grown to midlog phase in brain-heart infusion broth, washed, resuspended in saline, and injected i.v. (2 × 10^7^ CFU). Mice received two doses of mirtazapine or vehicle (20 mg/kg i.p.) 24 h and 2 h prior to infection with *S. aureus.*

### Assessment of Bacterial Load

Organs were harvested, weighed, and homogenized. Samples were filtered through a 100 μm strainer, centrifuged (2,500 rpm for 5 min, 4°C), and resuspended in PBS (1 ml). A series of 10-fold dilutions were performed in PBS containing chloramphenicol (1 mg/ml); 10 ul of each was plated on BHI agar plates and incubated at 37°C overnight.

### Statistical Analysis

Data are presented as mean ± SEM. Normally distributed data was assessed using the Grubbs’s test to identify and exclude potential statistical outlier data points. Student’s t-test was used unless otherwise indicated with significance set at p < 0.05.

## Results

### Mirtazapine Rapidly Activates Resident Hepatic Macrophages but Does Not Alter Pathogen Capture Abilities

**Figure 1:**
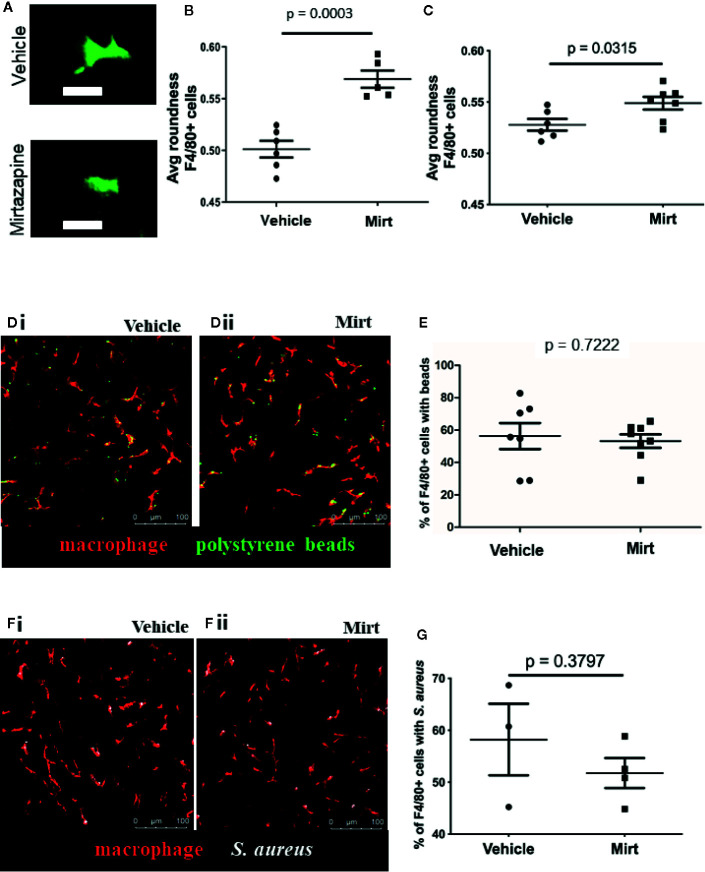
Assessment of F4/80+ cell shape in the liver by IVM. **(A)** Representative images from IVM showing the cell shape of F4/80+ cells in 3D reconstruction (vehicle-treated = top, mirtazapine-treated = bottom). **(B, C)** Quantification of IVM images 1.5 h **(B)** and 24 h **(C)** post-mirtazapine measuring average cell roundness (scale = 0➔1, where 1 is complete roundness). **(D)** Representative images of polystyrene bead capture in the livers of vehicle-treated **(Di)** and mirtazapine-treated **(Dii)** mice (macrophage labeled red; beads are green). **(E)** Quantification of the percentage of F4/80+ cells with bound beads in vehicle-treated, 24 h mirtazapine-treated animals. **(F)** Representative images of *S. aureus* particle capture in the livers of vehicle- **(Fi)** and mirtazapine-treated **(Fii)** mice (macrophage labeled red; *S. aureus* are white). **(G)** Quantification of the percentage of F4/80+ cells with captured *S. aureus* particles in vehicle-treated, 24 h mirtazapine-treated animals. Each dot represents an average of 5 FOV/mouse. Data are shown as mean +/− SEM, n = 5–6 animals/group. Scale bar = 20 µm in **(A)**; 100 µm in **(C)**.

IVM was performed on mice at 1.5 h post-injection with vehicle or mirtazapine. IVM clearly shows F4/80^hi^ macrophages within hepatic sinusoids (KCs) dynamically retracting their cellular pseudopods and changing morphology (**Figures 1A, B**). Measurement of cell “roundness,” a mathematical measure of how circular a cell is, demonstrates a significant shift in cell shape a readout that has been used both *in vitro* and *in vivo* to indicate changes in macrophage phenotype during an inflammatory response (19–21). Given the marked shift in cell morphology at 1.5 h post-mirtazapine, IVM was also performed 24 h post-mirtazapine in order to assess how long morphological changes persist. Although still significantly more round in mirtazapine *vs* vehicle treated animals, KCs had started to shift back to a less rounded phenotype by 24 h post-mirtazapine (**Figure 1C**, **Supplemental Movie 1**). The early marked shift in cellular morphology clearly demonstrates rapid activation of liver macrophages following mirtazapine treatment.

We next assessed whether mirtazapine-induced cellular activation and associated shape changes would affect the capacity of KCs to capture particles from the circulating blood (22). To test this, fluorescent 1 µm polystyrene beads were injected intravenously and the capacity of KCs to catch beads flowing through liver sinusoids was assessed, in real-time, utilizing IVM (using our previously published methods) (16). Fluorescent beads are commonly used as a pathogen surrogate to assess macrophage capture and/or phagocytosis (23–25). Results show that sinusoidal KCs have a similar capacity to capture beads from the hepatic circulation in both mirtazapine and vehicle treated groups (**Figures 1D, E**). As beads are inert particles, we confirmed our results using live *S. aureus* particles to rule out any pathogen-specific discrepancies. Although slightly more variable, *S. aureus* capture was not different between treatment groups (**Figures 1F, G**).

### Mirtazapine Alters the Liver Macrophage Response to Infection

**Figure 2:**
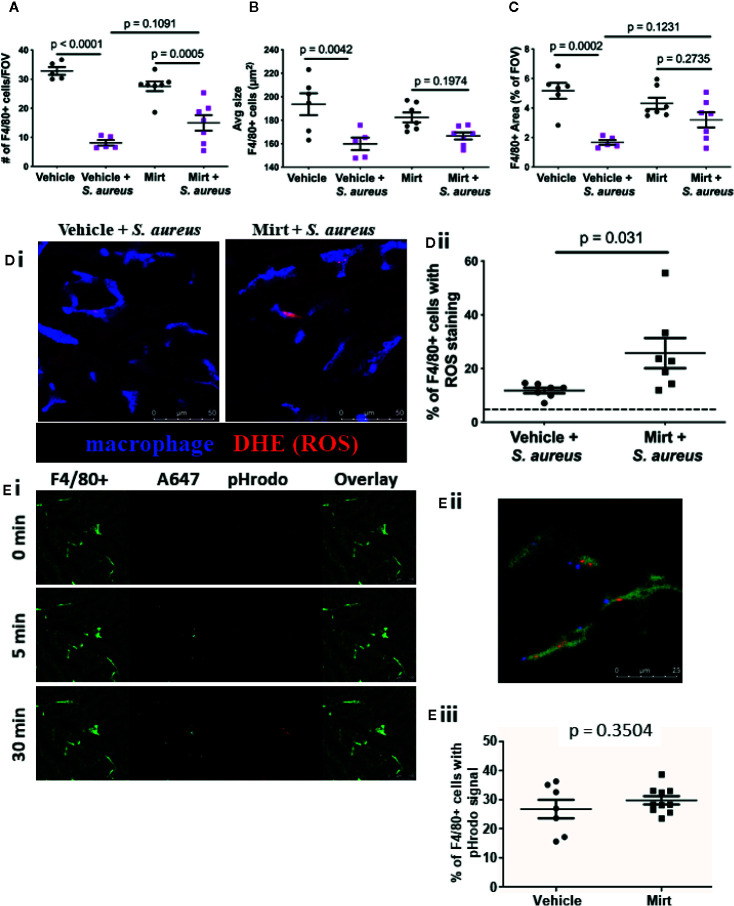
Macrophage response to S. *aureus * infection in mirtazapine treated mice. Measurement of the number of F4/80+ cells per FOV **(A)** F4/80+ cell size, **(B)**, and the total area covered by F4/80+ cells per FOV in mirtazapine-treated animals with or without S. aureus infection, compared to controls **(C)**. **(Di)** Representative IVM images of vehicle-treated (left) and mirtazapine-treated (right) mice 1h after intravenous injection of S. *aureus* [macrophage labelled blue; dihydroethidium (DHE) ROS probe is red]. **(Dii)** Quantification of percentage of DHE+ F4/80 cells, dashed line shows threshold level of ROS in uninfected controls (average of 5 FOV/animal). **(Ei)** Representative images of mouse liver at various times (0, 5, and 30 min) following injection of AF647-labelled pHrodo bioparticles. All bioparticles are visible in the AF647 (cyan) channel whereas those present in an acidified vesicle are also labelled in red (macrophage labelled bright green; hepatocyte autofluoresce in dark green). **(Eii)** Higher magnification of macrophage with captured bioparticles. **(Eiii)** Quantification of IVM images identifying the percentage of F4/80+ cells that contain positive pHrodo signal (red) (average of 5 FOV/animal). Data are shown as mean +/- SEM, n = 5-10 animals/group. Scale bar = 50 µm in **(Di)**; 25 µm in **(Eii)**; 100 µm in **(Ei)**.

Though the capacity for pathogen capture was unaltered by mirtazapine treatment, it is important to note that target capture alone does not directly assess the efficiency and effectiveness of phagocytosis or activation of downstream bacterial killing mechanisms, such as oxidative burst, within the cell. To directly address this question, we examined KC function during a systemic intravascular bacterial infection using a model of i.v. injection of a pathogenic strain of *S. aureus* (USA300). In animals infected with *S. aureus*, a significant reduction in the number of F4/80+ macrophages was noted within the liver (**Figure 2A**). This observed cell loss was not prevented in mirtazapine-treated animals. Moreover, liver macrophages in both vehicle-treated and mirtazapine-treated mice showed a significant reduction in cell size following *S. aureus* infection (**Figure 2B**, **Supplemental Movie 2**). Although the observed cell loss and the reduction in cell size were significant following *S. aureus* infection of mirtazapine-treated mice, these alterations to liver macrophages were less apparent than those documented in vehicle-treated mice. As such, F4/80+ cells occupied a significantly lower proportion of each liver imaging FOV in vehicle treated, but not in mirtazapine-treated mice following infection (**Figure 2C**). Overall, infection of mirtazapine-treated animals resulted in maintenance of liver coverage by intravascular macrophages post-infection, as compared with vehicle-treated animals.

To further address the impact of mirtazapine on the ability to KC to respond to infection, *S. aureus* was injected with a superoxide indicator (dihydroethidium; DHE) to visualize the ability of KCs to ingest (i.e. phagocytize) bacteria, and subsequently activate reactive oxygen species (ROS) generation within the cell. Generation of ROS is a well-defined bacterial killing mechanism within macrophages. Indeed, we found an increase in the percentage of KCs that contained ROS signal in vehicle treated mice that had received *S. aureus* (**Figure 2D**), compared to uninfected controls. Interestingly, we found an even higher percentage of KCs that were positive for ROS signal following *S. aureus* infection in mice that had been pretreated with mirtazapine, suggesting that observed liver macrophage activation (shape change) is also associated with an enhanced capacity to produce ROS.

Given that mirtazapine treatment enhanced KC capacity to generate ROS, we hypothesized that mirtazapine might also enhance other intracellular killing mechanisms within KCs. Following phagocytosis of bacteria, macrophages fuse the phagosome containing the pathogen, with a lysosome, to form the phagolysosome. Following this fusion, the pH within the phagolysosome gradually decreases to activate lysosomal enzymes in an effort to destroy the ingested bacteria. Direct visualization of this process *in vivo* is accomplished using IVM and a pH-sensitive probe, and has been shown to occur within 30 min after bacterial administration (16). To visualize both bacterial capture, phagocytosis and subsequent phagolysosomal maturation by IVM, pHrodo *S. aureus* bioparticles counter-labeled with AlexaFluor 647 were administered i.v. (16). This method allows for *S. aureus* bioparticles to be injected and visualized (AlexaFluor 647 signal), and after phagocytosis and internalization, the maturation of the phagosome can be tracked as it progresses to form a phagolysosome, identified as the pHrodo molecule becomes fluorescent in the presence of a drop in vesicular pH (**Figure 2Ei**). We found that mirtazapine had no impact on this process, with IVM quantification demonstrating the same percentage of KCs positive for pHrodo signal in mirtazapine treated animals compared to vehicle treated controls following infection (**Figure 2Eii**). The observation that mirtazapine has no effect on phagolysosomal maturation suggests that enhanced ROS production in KCs in mirtazapine-treated, compared to vehicle treated mice after *S. aureus* infusion, is a specific change, and does not reflect an overall hyper-responsiveness in KCs following mirtazapine treatment.

### Mirtazapine Treatment Results in Reduced Neutrophil Recruitment to the Liver In Response to Infection

**Figure 3:**
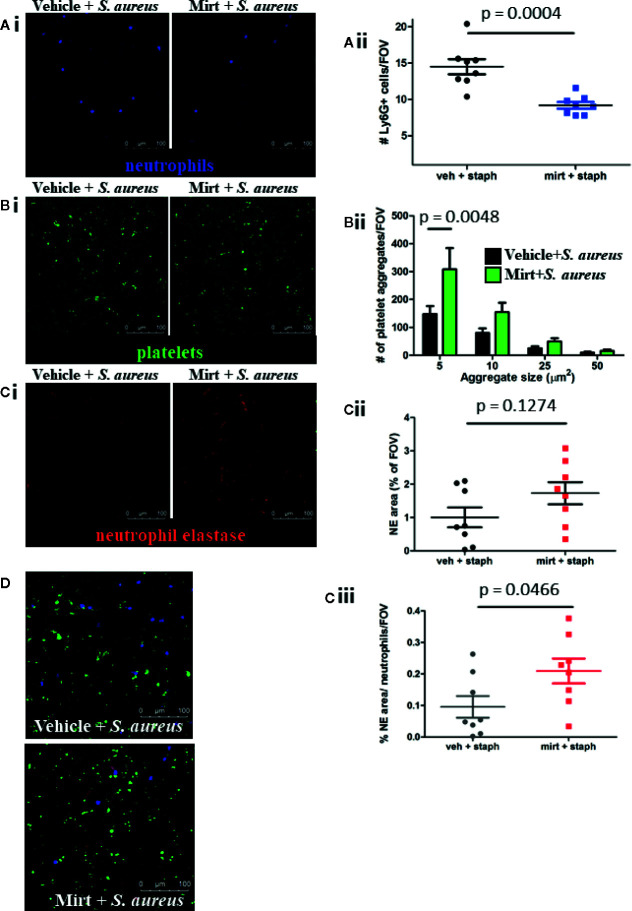
IVM analysis of the neutrophil and platelet response 24 h post *S. aureus* infection in mirtazapine-treated mice. **(Ai–Ci)** Representative images of mice treated with vehicle (left) or mirtazapine (right) and infected with *S. aureus* for 24 h. Quantification of Ly6G+ cells **(Aii)**, number of platelet aggregates equal-to or greater-than indicated sizes **(Bii),** area of neutrophil elastase staining **(Cii),** and area of neutrophil elastase staining normalize to the number of neutrophils **(Ciii)** per field of view (average of 5 FOV/mouse). **(D)** Representative images of neutrophil, platelet, and elastase staining overlaid from vehicle or mirtazapine treated animals infected with *S. aureus* (neutrophils labeled blue; platelets labeled green; neutrophil elastase labeled red). Data are shown as mean +/− SEM, n = 8 animals/group. Scale bar = 100 µm.

Given the enhanced ROS signal found in liver macrophages after mirtazapine treatment, we wondered how mirtazapine would affect the ensuing inflammatory response in the liver after bacterial challenge. Previous work has shown that peak neutrophil recruitment to the liver occurs 4 h after bacterial infection and these neutrophils provide a scaffold facilitating platelet aggregation leading to the production of neutrophil extracellular traps (NETs) (26). NETs are potent innate immune effectors, able to ensnare and kill pathogens in sticky webs of DNA decorated with cytotoxic and antimicrobial granule-derived proteins (e.g. neutrophil elastase). Following *S. aureus* infection of mirtazapine-treated mice, we observed reduced neutrophil recruitment to the liver, as compared to infection of vehicle-treated animals (**Figure 3A**). Surprisingly, despite fewer adherent neutrophils on which to bind, mirtazapine-treatment leads to enhanced platelet aggregation within the liver following *S. aureus* infection (**Figure 3B**). Although neutrophil recruitment is reduced in mirtazapine-treated animals, enhanced platelet aggregation ensures that overall NET production is not inhibited (**Figures 3Ci, Cii**). Interestingly, when NET production is normalized to the number of neutrophils in the liver, it becomes apparent that mirtazapine-treatment leads to enhanced NET production on a per-neutrophil basis following *S. aureus* infection (**Figure 3Ciii**). To determine if mirtazapine mediates its effects directly on the neutrophil or the platelet, we assessed neutrophil activation following *in vitro* stimulation with mirtazapine, following *in vivo* treatment, and assessed platelet activation following *in vitro* stimulation with mirtazapine (**Supplemental Figure 1**). Thus, despite reduced neutrophil recruitment, mirtazapine-treated animals continued to support robust platelet aggregation and NET production within the liver following infection (**Figure 3D**). Furthermore, we found that during *S. aureus* sepsis, the mirtazapine-related increase in platelet aggregate formation and neutrophil NET generation was associated with increased serotonin levels within the circulation, compared to septic mice that received vehicle (**Supplemental Figure 2**).

### Mirtazapine-Treatment Reduces Infection-Induced Liver Damage While Preserving Pathogen Clearance

**Figure 4:**
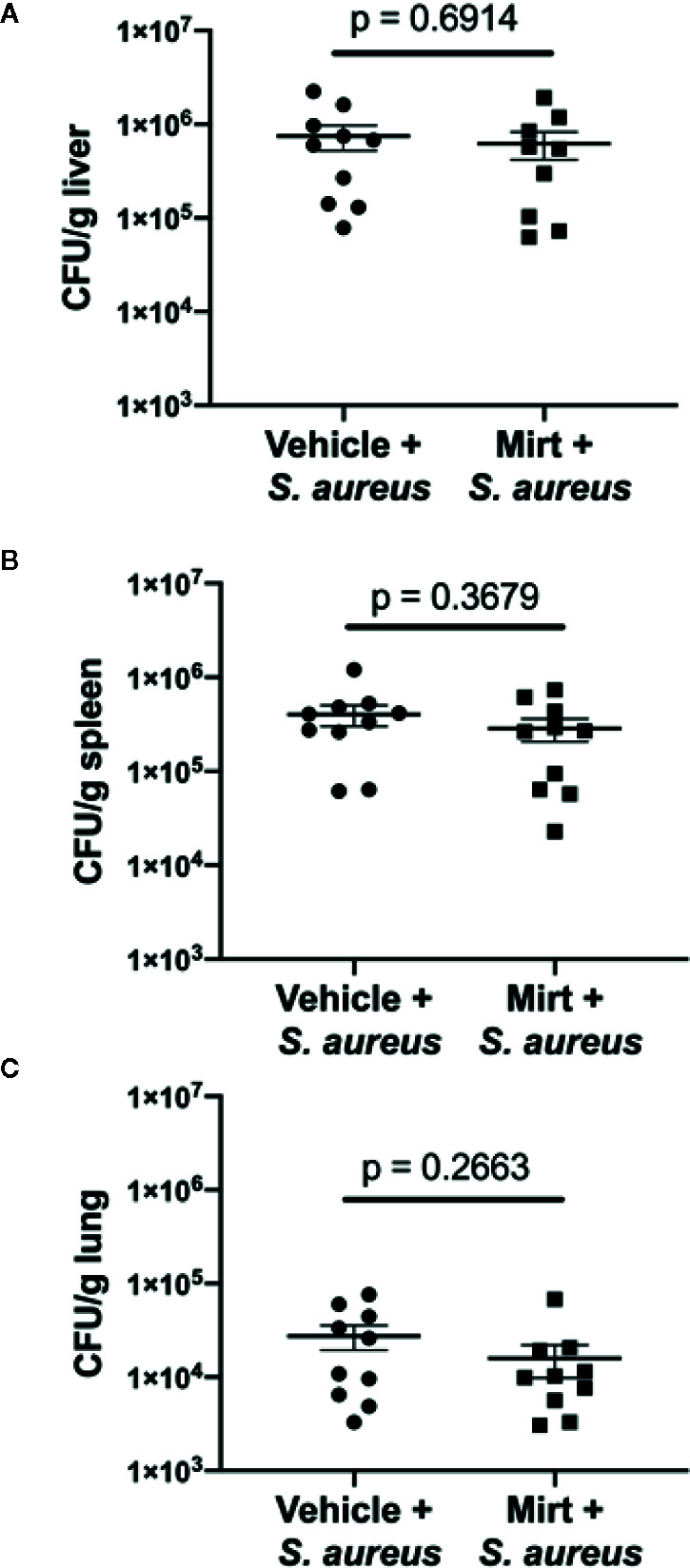
Measurement of bacterial dissemination 24 h post-infection in mirtazapine treated mice. Bacterial load (colony forming units per gram of tissue [CFU/g]) in **(A)** liver **(B)** spleen and **(C)** lung 24 h post-infection with *S. aureus.* Data are shown as mean +/− SEM, n = 10 animals per group.

Given the observed alterations in innate immune cell function within the liver of mirtazapine-treated animals, we next addressed the overall capacity of the host to respond to, contain, and limit dissemination of an intravenous bacterial infection. Mice were treated with mirtazapine (-24 h and -1 h) prior to infection with *S. aureus*, and at 24 h post-infection tissues were harvested for assessment of bacterial loads. Despite significant alterations in KC ROS potential and reduced neutrophil recruitment to the liver, no significant differences were observed in the bacterial loads measured in the liver, lung, or spleen (**Figure 4**). Moreover, serum ALT levels were also similar in these groups of mice (**Supplemental Figure 3**). This data strongly indicate that despite reduced neutrophil recruitment to the liver and alterations to macrophage phenotype, there is no increased bacterial dissemination in mirtazapine-treated animals at this acute timepoint.

**Figure 5:**
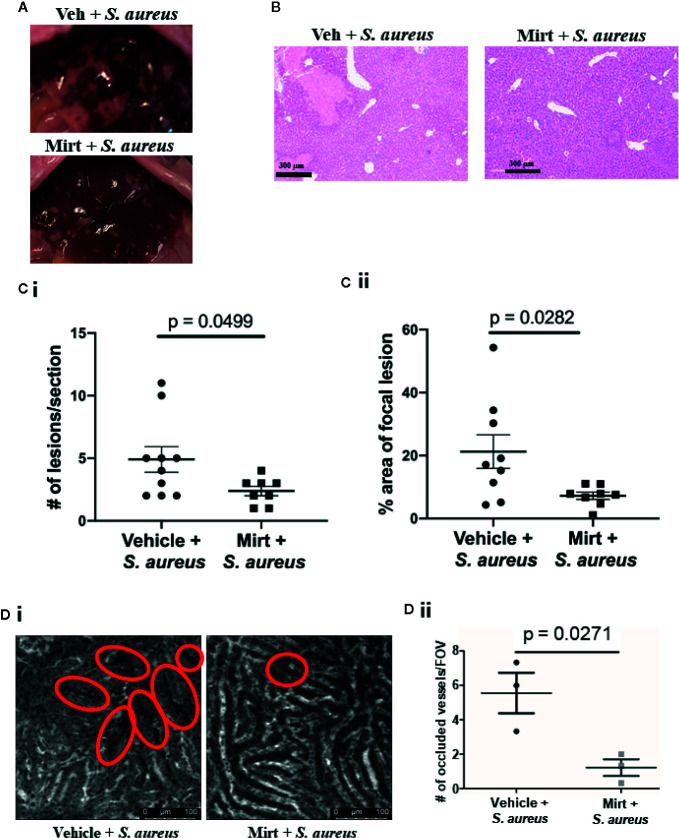
Measurement of liver damage 24 h after S. aureus infection in mirtazapine treated mice. **(A)** Representative images of livers collected from mice treated with vehicle or mirtazapine 24 hrs post S. aureus infection. **(B)** H&E stained liver sections 24 hrs post S. aureus infection of vehicle- or mirtazapine-treated animals. Quantification of the **(Ci)** number and the **(Cii)** area (% of a field of view occupied by necrotic tissue) of observed histological focal necrotic lesions in mice infected with S. aureus. **(Di)** Representative IVM images demonstrating vascular perfusion 24 hrs post S. aureus infection of vehicle- or mirtazapine-treated animals as visualized following i.v. injection of FITC-dextran. Sinusoids with non-perfused regions are denoted by the red circles. **(Dii)** Quantification of the number of occluded sinusoids per FOV. Data are shown as mean +/- SEM, n=3-10 animals/group. Scale bar = 300 µm in **(A)**; 100 µm in **(Di)**.

The inflammatory innate immune response to bacterial infection is often associated with significant collateral host tissue damage (26, 27). To determine if mirtazapine-treatment impacted host tissue damage following infection we collected plasma and liver tissue 24 h post *S. aureus* infection in vehicle and mirtazapine-treated animals. Observing gross anatomical differences in the livers of mirtazapine and vehicle pre-treated infected mice (**Figure 5A**), we sought to quantify changes in lesions by histology. Tissue sections stained with hematoxylin and eosin revealed less pronounced necrosis within the liver following *S. aureus* infection in mirtazapine-treated animals (**Figure 5B**). Quantification of this pathology revealed both fewer necrotic lesions (**Figure 5Ci**) and smaller lesions (**Figure 5Cii**) in the livers of mirtazapine-treated mice. The presence of smaller lesions in the mirtazapine-treated animals led us to assess vascular perfusion following *S. aureus* infection. Previous work has shown that infection-induced coagulation leads to vascular occlusion and associated tissue damage (27, 28). Infection of mirtazapine-treated animals resulted in fewer occluded sinusoids (**Figure 5D**), confirming reduced liver damage was associated with improved perfusion and less infection-induce coagulation.

## Conclusions

Mirtazapine treatment has a beneficial impact on immune-mediated liver disease in patients and animal models (10, 12). We have previously demonstrated a suppressive effect of mirtazapine on liver injury in a mouse model of immune-mediated hepatitis, mediated in part through attenuation of hepatic innate immune responses and activation of KCs (12). Therefore, we speculated that mirtazapine may adversely impact systemic bacterial capture and killing, processes critically reliant on KC activation and function (14). We found that mirtazapine treatment rapidly induces KC shape changes (within 1.5 h), with cells taking on a more rounded and activated phenotype. However, in contrast to our initial speculation, we found that mirtazapine treatment did not alter the ability of KCs to capture pathogens from the circulation, but rather, enhanced intracellular ROS generation by these KCs. Although systemic bacterial dissemination after *S. aureus* injection was not altered post-mirtazapine treatment, hepatic abscess size was significantly reduced. These findings suggest that mirtazapine induces changes in the liver (macrophage area, enhanced ROS, reduced neutrophil recruitment, efficient NETosis) to enable efficient pathogen clearance in the presence of reduced inflammatory infiltrate, resulting in reduced immune-mediated liver damage (10, 12) following bacterial infection.

Mirtazapine is a tetracyclic piperazinoazepine that exhibits a complex pharmacology, having both central and peripheral effects (29). Specifically, mirtazapine acts as a 5HT2_A_ receptor antagonist, 5HT2_C_ receptor inverse agonist, and an antagonist for 5HT_3_ and histamine (H1) receptors (29, 30). Therefore, in keeping with its wide range of receptor activity, mirtazapine has been increasingly used clinically to treat a broad range of symptoms including depression, anxiety, and insomnia (29). However, in addition to its effects on symptoms, there is evidence that mirtazapine also affects systemic (30–32) and hepatic (12, 33) immunity.

KCs play a key role as sentinels within the bloodstream during sepsis (34). For example, acute staphylococcal infection results in the rapid sequestration of most bacteria by KCs, with associated KC activation and pathogen killing (13, 35). Macrophages, including KCs, exist within a spectrum of activation states including M1-like (proinflammatory) and M2-like (anti-inflammatory, repair) (36). In response to cytokines produced during infection and other danger signals, macrophages shift towards an M1phenotype, producing reactive species (e.g. ROS) (16, 37) and inflammatory cytokines to combat invading pathogens (36, 38, 39). Moreover, it is clear that macrophage shape changes are also associated with different activation phenotypes (38). Specifically, M1 macrophages appear flatter and more rounded, whereas M2 macrophages are elongated (38). KCs exist within the hepatic sinusoids as flattened cells, with cellular processes extending along the sinusoidal endothelium. However, after mirtazapine treatment KCs rapidly shape change, assuming a more rounded appearance with retracted cellular processes, suggestive of an M1 phenotype. However, despite these pronounced shape changes, we found no significant impact of mirtazapine on the capture of intravenously administered beads by KCs (a validated surrogate for bacterial capture) (40), compared to vehicle treated mice.

After capture by KCs, bacteria such as *S. aureus* are internalized within phagosomes and become acidified and facilitate the generation of ROS, with associated bacterial killing (41). We found a similar degree of *S. aureus*-induced phagosome acidification in KCs in mirtazapine and vehicle treated mice, but found significantly augmented KC production of ROS in mirtazapine *vs* vehicle treated mice after intravenous injection of *S. aureus*. These findings are consistent with our observations of mirtazapine-induced KC shape changes, suggesting a shift in KCs towards a more proinflammatory, antibacterial phenotype after mirtazapine treatment.

How might mirtazapine drive a shift in KC phenotype? Macrophages express numerous receptors for neurotransmitters, including serotonin receptors, and serotonin induces phenotypic shifts in both human and murine macrophages. Specifically, serotonin skews macrophage polarization towards an M2 phenotype *in vitro*, through activation of 5HTR_2B_ and 5HTR_7_ receptor subtypes (42). Consistent with this observation, serotonin suppresses ROS production in macrophages *in vitro* (43). Interestingly, activation of 5HTR_7_ receptors in murine macrophage-derived dendritic cells causes cellular elongation and lengthening of cellular processes (44); a cellular shape change associated with an M2 phenotypic shift in macrophages (38). In contrast, serotonin shifts murine alveolar macrophages towards an M1 phenotype *via* stimulation of 5HT2_C_ receptors (45). Given mirtazapine’s complex interplay with a number of serotonergic receptors, the balance of these effects could significantly alter KC phenotype *in vivo* and explain our IVM observations.

Platelets are a rich source of serotonin which in turn contributes to the regulation of hepatic physiological and pathophysiological responses (46, 47). Although mirtazapine alone did not appear to impact plasma serotonin levels, there was a clear priming event such that subsequent bacterial infection resulted in a significant increase in serotonin levels over what was observed in infected vehicle-treated mice. Moreover, our findings indicate enhanced platelet recruitment and aggregation in the liver of mirtazapine-treated mice during *S. aureus* sepsis. Activated platelets release serotonin and augment neutrophil NET release (48, 49), and we now show that mirtazapine treatment augmented both platelet aggregate formation and neutrophil NET release.

During *S. aureus* sepsis, KCs effectively function as an “infection bottleneck,” as depletion of KCs in mice increases *S. aureus* susceptibility, enhances systemic spread, and strikingly increases bacterial burden and abscesses within the liver (50). In contrast, hepatic recruitment of neutrophils appears to be most important for preventing systemic *S. aureus* dissemination throughout the body (50). In our current study, we found that mirtazapine treatment did not alter systemic spread of *S. aureus*, or the absolute number of bacteria within the liver (as CFUs), but did significantly reduce the overall *S. aureus* abscess burden within the liver post-bacteria injection (as % necrotic area within the liver). These findings suggest that the phenotypic changes in KCs induced by mirtazapine are associated with an enhancement in KC-induced attenuation of *S. aureus*-mediated abscess expansion. This effect may be explained, at least in part, by the reduction in neutrophil recruitment to the liver post-*S. aureus* administration in mice that received mirtazapine, compared to those that received vehicle. Neutrophils play an important effector role in pathogen elimination through phagocytosis, production of toxic metabolites, and release of proteolytic enzymes (51, 52). However, these neutrophil-mediated actions can also lead to bystander injury in the liver (52–55), and attenuation of neutrophil recruitment contributes to limiting infection-induced liver damage. Importantly, mirtazapine-treated animals demonstrated less vascular occlusion and increased sinusoidal perfusion following *S. aureus* infection. We have previously demonstrated sinusoidal occlusion as the key driver in localized liver damage following infection (27, 28). Thus, is it likely the reduced liver necrosis observed in mirtazapine-treated animals is associated with reduced vascular occlusion. Interestingly, reduced hepatic abscess size in mirtazapine treated septic mice was not associated with a reduction in serum ALT levels compared to vehicle treated septic mice. The reason for this is unclear, however discrepancies between ALT levels and degree of hepatic necrosis have been described in both human and animal models of liver disease (56–59).

In summary, we have identified an important beneficial role for mirtazapine in the regulation of hepatic *S. aureus* dynamics, by altering KC phenotype to enhance KC production of ROS in response to *S. aureus* infection and to decrease neutrophil recruitment, hepatic abscess growth, and associated tissue destruction. The overall reprogramming of liver innate immunity appears highly coordinated and integrated with enhanced bactericidal potential of KCs offsetting reduced recruitment of neutrophils resulting in an overall attenuation of inflammation-induced liver damage. These findings suggest a potential beneficial effect of mirtazapine in preventing some complications associated with *S. aureus* sepsis and warrants further exploration.

## Data Availability Statement

The raw data supporting the conclusions of this article will be made available by the authors, without undue reservation.

## Ethics Statement

The animal study was reviewed and approved by University of Calgary Animal Care Committee.

## Author Contributions

RD performed experiments, analyzed the data, prepared figures, and wrote the manuscript. WA performed experiments and contributed to the generation of the manuscript. CJ and MS conceived, designed, and supervised the research, analyzed data, and wrote the manuscript. All authors contributed to the article and approved the submitted version.

## Funding

This study was supported by grants from the Canadian Foundation for Innovation, Alberta Innovates and Advanced Education, the Cal Wenzel Family Foundation Chair in Hepatology (MS), and the Canadian Institutes in health Research Team Inflammation grant. RD is funded by the Canadian Liver Foundation and the University of Calgary Faculty of Graduate Studies Indigenous Graduate Award. CJ is supported by the Canada Research Chairs program.

## Conflict of Interest

The authors declare that the research was conducted in the absence of any commercial or financial relationships that could be construed as a potential conflict of interest.
