# Supplementary material for: The Antidepressant Mirtazapine Activates Hepatic Macrophages, Facilitating Pathogen Clearance While Limiting Tissue Damage in Mice
Source: Front Immunol. 2020 Nov 3;11:578654. doi: 10.3389/fimmu.2020.578654 (PMC7673391; doi:10.3389/fimmu.2020.578654)
Supplement: Supplementary file 1 [file Table_1.docx]

Supplementary Material

# Supplemental Table 1

| Antibody/Probe | Clone | Supplier |
| --- | --- | --- |
| Anti-Ly6G | 1A8 | Biolegend |
| Anti-F4/80 | BM8 | Biolegend |
| Anti-CD41 | HMα2 | Biolegend |
| Anti-Neutrophil Elastase | M18 | Abcam |
| Fluorescent Microspheres | Fluoresbrite YG 1µm Microspheres | Polysciences Inc. |
| Dihydroethidium | - | ThermoFisher |
| pHrodo™ Red S. aureus Bioparticles™ | - | ThermoFisher |

# Supplementary Movies

**Supplementary Movie 1.** Intravital visualization of liver macrophage in vehicle-treated and mirtazapine-treated mice. Macrophage (F4/80+ cells) are labelled in green, blood vessels are labelled in blue.

**Supplementary Movie 2.** Intravital visualization of liver macrophage 24 hrs following i.v. *S. aureus* infection in vehicle-treated and mirtazapine-treated mice. Macrophage (F4/80+ cells) are labelled in pink, blood vessels are labelled in blue.

# Supplementary Methods

*Flow cytometry*: Blood from untreated, vehicle-, and mirtazapine-treated animals were collected in 4% sodium citrate (Sigma-Aldrich). For *in vitro* stimulation, blood from untreated mice was incubated with vehicle or mirtazapine (10 μM) for 30 mins. To remove red bloods cells, 1X RBC lysis buffer (Biolegend, cat 420302) was added for 5 mins and samples were centrifuged at 800 x g for 3 mins. Cells were washed in PBS and stained with fixable viability dye for later removal of dead cells (ThermoFisher, L34973). After PBS wash, cells were stained with mAb diluted in FACS wash buffer (PBS, 1 mM EDTA, 2% FBS) for 30 min on ice, washed, and fixed with 4% formaldehyde fixation buffer (BD, Cat 554655). Cells were then washed and resuspended in 200 μl of cold FACS wash buffer. Analysis was performed on an Attune Acoustic Focusing Cytometer (Life Technologies, Carlsbad, California).

*ALT measurements*: Blood was collected from *S. aureus* infected mice that were pre-treated with mirtazapine (or vehicle) in a 1:10 volume of 4% sodium citrate (Sigma-Aldrich) via cardiac puncture. Samples were centrifuged at 1000 x g for 15 mins. Plasma was collected and sent to Calgary Lab Services for ALT assessment.

*Serotonin ELISA:* Blood was collected via cardiac puncture from mirtazapine (or vehicle) + *S. aureus* infected mice in a 1:10 volume of 4% sodium citrate (Sigma-Aldrich). After centrifugation (1000 x g for 15 mins), serotonin ELISA (ab133053) was performed according to manufacturer instructions.

**4 Supplementary Figures**

**
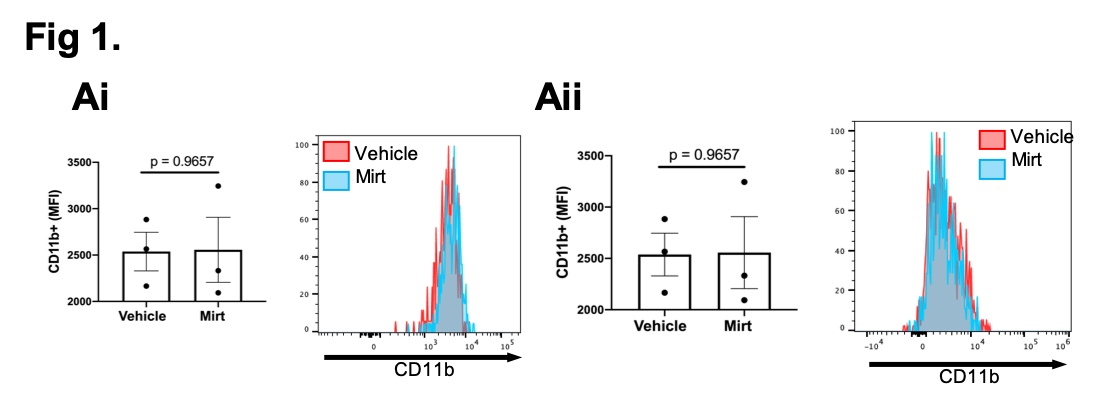
**

**
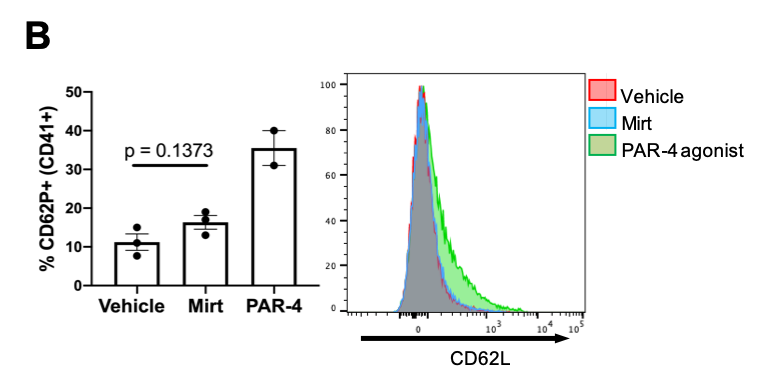
**

**Supplemental Fig 1.** Flow cytometric analysis of mirtazapine stimulated blood neutrophils *in vitro* (Ai) or *in vivo* (Aii). Samples were gated on Ly6G+ and analyzed for mean fluorescence intensity (MFI) of CD11b+. (B) Flow cytometric analysis of *in vitro* stimulated platelet rich plasma. Samples were gated on CD41+ and quantified as percentage of CD62P+ (PAR-4 agonist included as positive control).


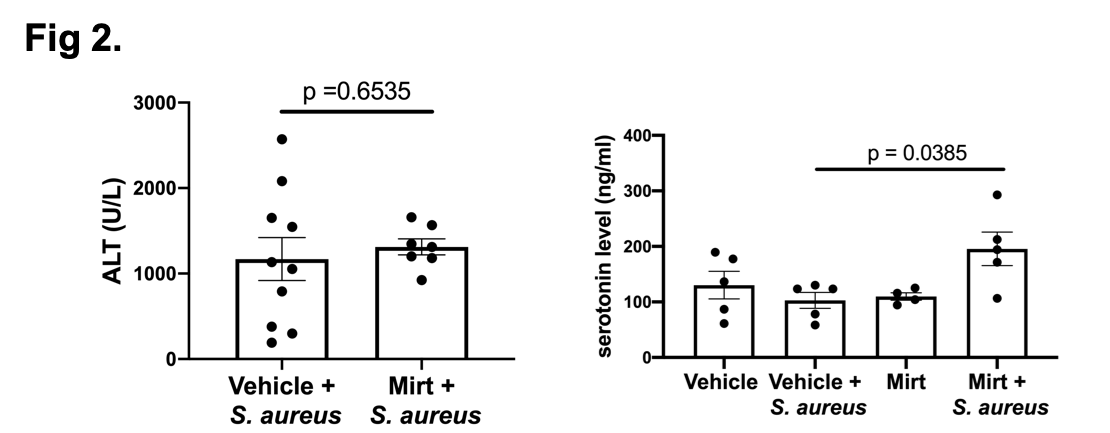

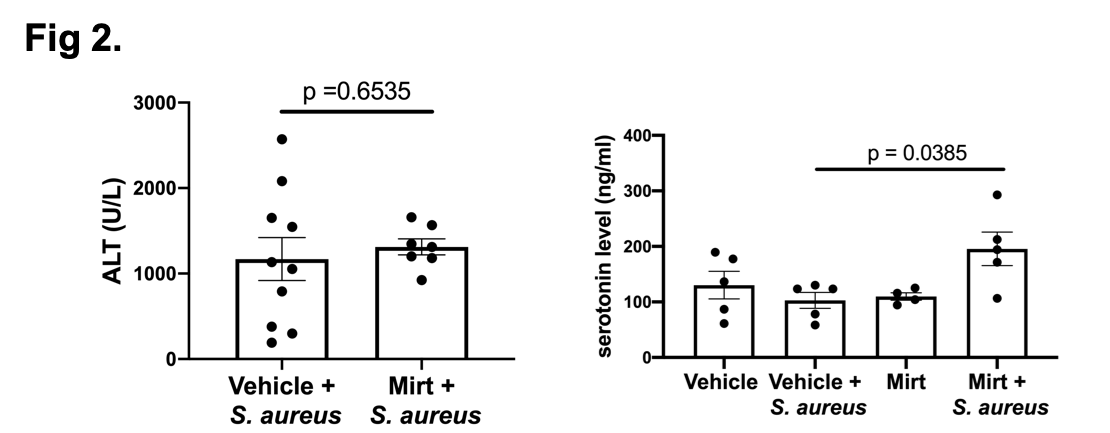


**Supplemental Fig 2.** Serotonin levels measured by ELISA 24 hrs post-*S. aureus* infection of vehicle- or mirtazapine-treated animals compared to uninfected controls. Data are shown as mean +/- SEM, n = 5 animals per group.


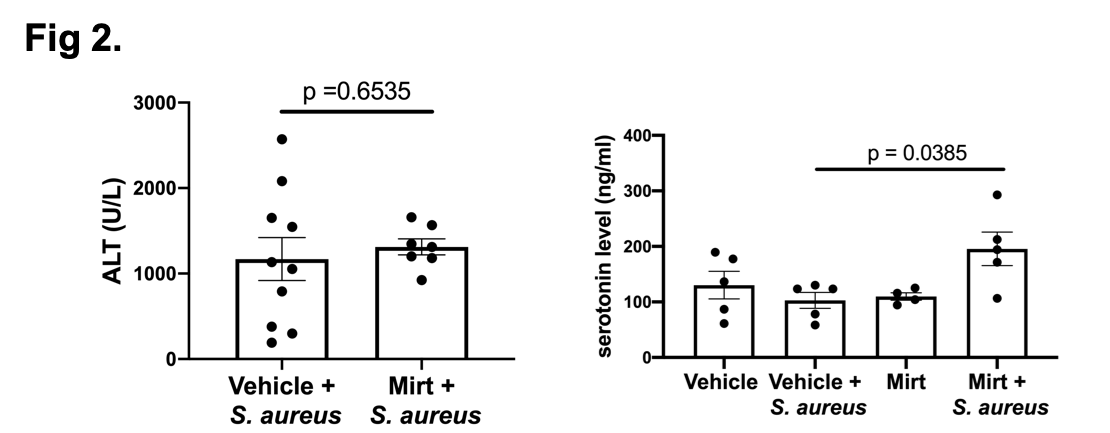


**3**

**Supplemental Fig 3.** ALT levels 24h post *S. aureus* infection of vehicle- or mirtazapine-treated animals (n=7-10 animals/group). Data are shown as mean +/- SEM, n = 5 animals per group.
